# Supplementary material for: Type-I IFNs in EAE drive synaptic transcriptional responses in neurons and astrocytes in the brain
Source: bioRxiv. 2026 Sep 25:2026.09.23.753833. Preprint. [Version 1] doi: 10.64898/2026.09.23.753833 (PMC13622232; doi:10.64898/2026.09.23.753833)
Supplement: Supplementary file 1 [file NIHPP2026.09.23.753833v1-supplement-1.pdf]

bioRxiv preprint doi: https://doi.org/10.64898/2026.09.23.753833; this version posted September 25, 2026. The copyright holder for this preprint (which was not certified by peer review) is the author/funder, who has granted bioRxiv a license to display the preprint in perpetuity. It is made available under a CC-BY 4.0 International license.

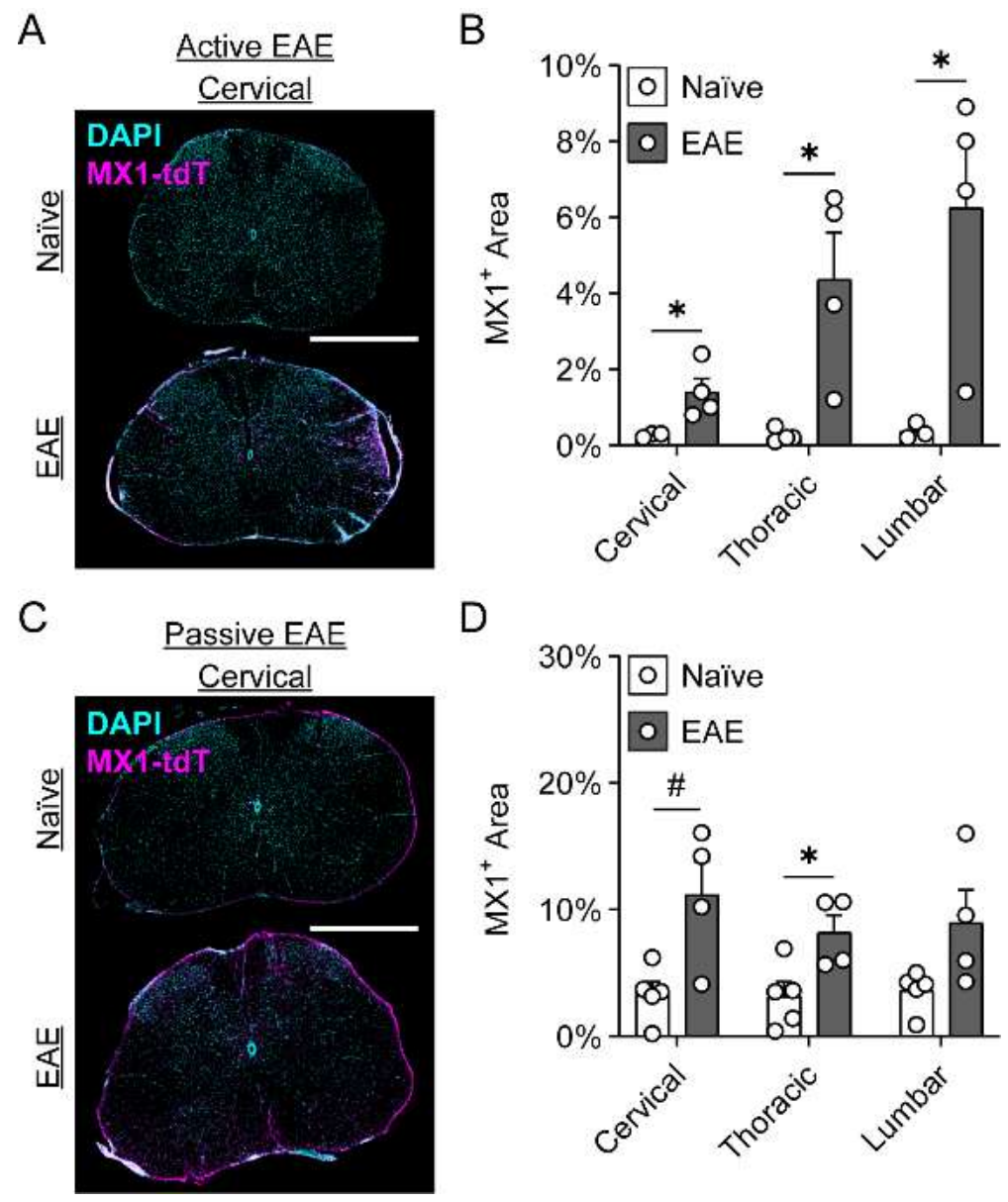

Figure S1. IFN signaling increases in the spinal cord at the onset of EAE symptoms. (A) Representative spinal cord sections from active EAE mice at 14 DPI. Scale bar = 1 mm. **(B)** Quantification of MX1-tdT expression in the spinal cord of active EAE mice. Main Effect of Treatment: $F(1, 6) = 18.01$, $P = 0.0054$. **(C)** Representative spinal cord sections from passive EAE mice at 12 DPI. Scale bar = 1 mm. **(D)** Quantification of MX1-tdT expression in the spinal cord of passive EAE mice. Data shown are mean ± SEM. $^{*}P < 0.05$; $^{\#}P < 0.10$.

bioRxiv preprint doi: https://doi.org/10.64898/2026.09.23.753833; this version posted September 25, 2026. The copyright holder for this preprint (which was not certified by peer review) is the author/funder, who has granted bioRxiv a license to display the preprint in perpetuity. It is made available under a CC-BY 4.0 International license.

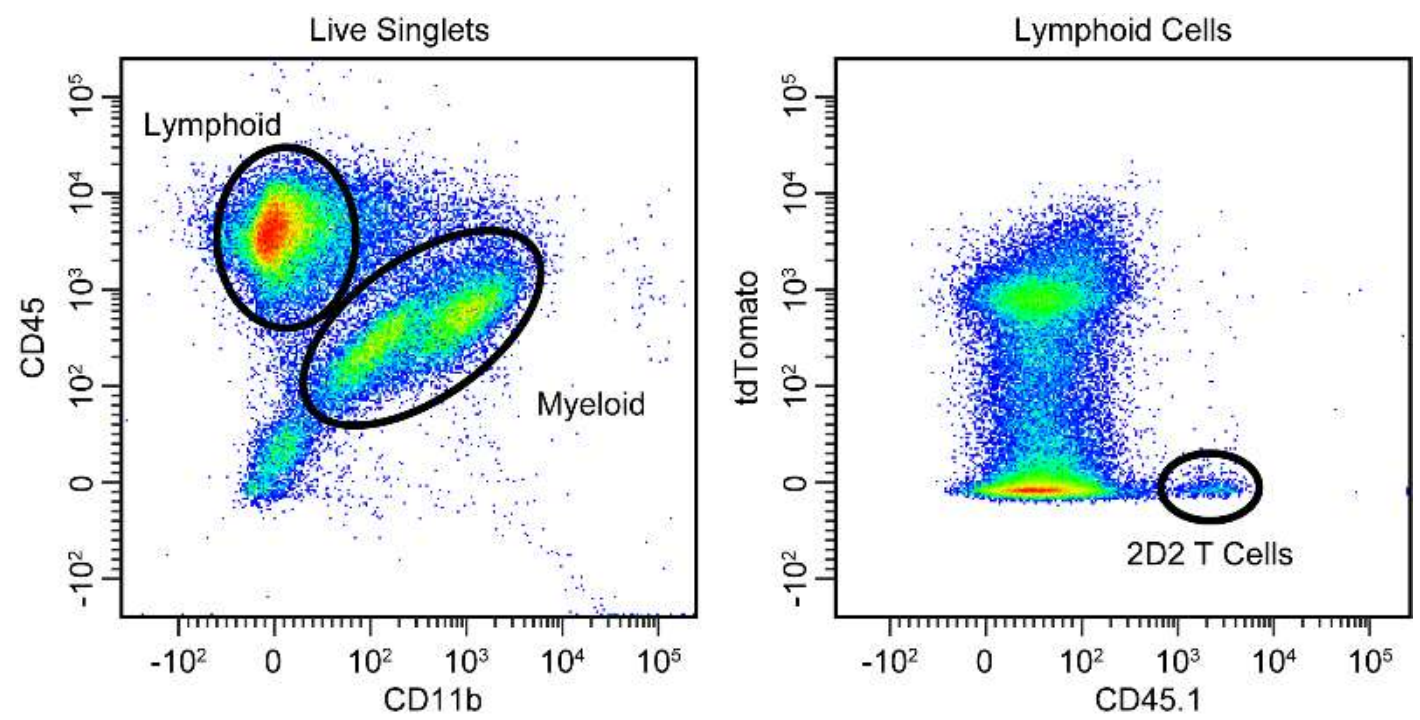

Figure S2. Representative flow cytometry gating strategy to identify MX1-tdT⁺ 2D2 T cells. Lymphoid cells were defined as CD45⁺/CD11b⁻ populations. 2D2 cells were defined as lymphoid cells that were tdT⁻/CD45.1⁺.

bioRxiv preprint doi: https://doi.org/10.64898/2026.09.23.753833; this version posted September 25, 2026. The copyright holder for this preprint (which was not certified by peer review) is the author/funder, who has granted bioRxiv a license to display the preprint in perpetuity. It is made available under a CC-BY 4.0 International license.

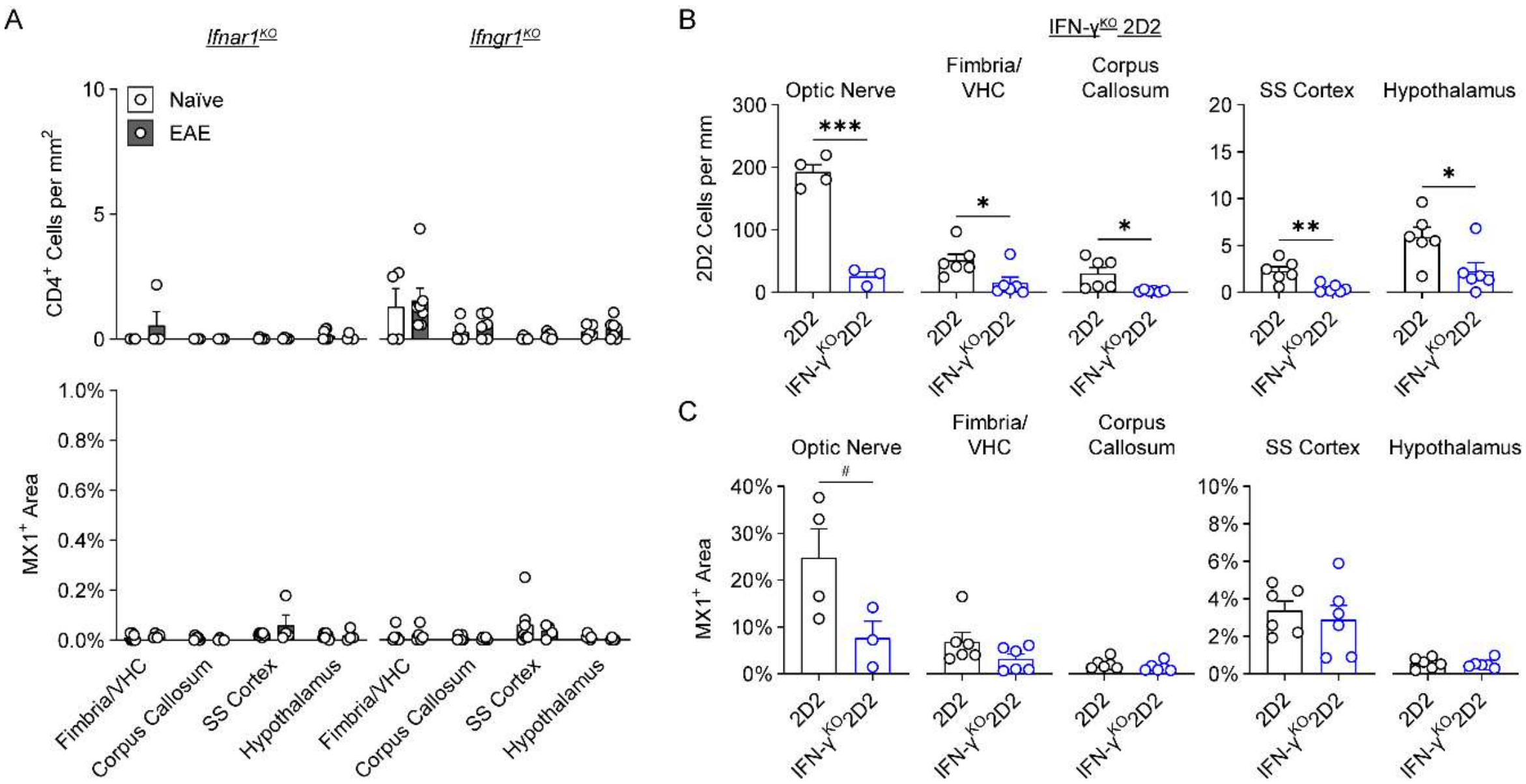

Figure S3. Attenuation of IFN-signaling in EAE prevents the infiltration of CD4⁺ T cells into the brain. (A) Quantification of CD4⁺ T cell infiltration and MX1-tdT expression in various white matter and gray matter regions of *Ifnar1^KO^* and *Ifngr1^KO^* mice with and without EAE induction. **(B&C)** Quantification of CD4⁺ T cell infiltration and MX1-tdT expression in various white matter and gray matter regions of mice that received 2D2 or IFN-γ^{KO} 2D2 T_h1 cells. Data shown are mean ± SEM. **P* < 0.05; ***P* < 0.01; ****P* < 0.001.
